# Insights into the Functions of eIF4E-Binding Motif of VPg in Potato Virus A Infection

**DOI:** 10.3390/v12020197

**Published:** 2020-02-11

**Authors:** Shreya Saha, Kristiina Mäkinen

**Affiliations:** Department of Microbiology and Viikki Plant Science Centre, University of Helsinki, 00014 Helsinki, Finland; Shreya.Saha@helsinki.fi

**Keywords:** potato virus A, potyvirus, viral protein genome-linked, eukaryotic initiation factor 4E/(iso)4E, potyvirus-induced RNA granules, RNA silencing, RNA silencing suppression

## Abstract

The interaction between the viral protein genome-linked (VPg) and eukaryotic initiation factor 4E (eIF4E) or eIF(iso)4E of the host plays a crucial role in potyvirus infection. The VPg of potato virus A (PVA) contains the Tyr-X-X-X-X-Leu-phi (YXXXLΦ) binding motif for eIF(iso)4E. In order to investigate its role in PVA infection, we substituted the conserved tyrosine and leucine residues of the motif with alanine residues in the infectious cDNA of PVA (PVA^VPgmut^). PVA^VPgmut^ RNA replicated in infiltrated leaves, but RNA accumulation remained low. Systemic infection occurred only if a reversion to wild type PVA occurred. VPg was able to stabilize PVA RNA and enhance the expression of *Renilla* luciferase (3’RLUC) from the 3’ end of the PVA genome. VPg^mut^ could not support either PVA RNA stabilization or enhanced 3’RLUC expression. The RNA silencing suppressor helper-component proteinase (HCPro) is responsible for the formation of PVA-induced RNA granules (PGs) during infection. While VPg^mut^ increased the number of PG-like foci, the percentage of PVA RNA co-localization with PGs was reduced from 86% to 20%. A testable hypothesis for future studies based on these results is that the binding of eIF(iso)4E to PVA VPg via the YXXXLΦ motif is required for PVA RNA stabilization, as well as the transfer to the RNA silencing suppression pathway and, further, to polysomes for viral protein synthesis.

## 1. Introduction

Plant–pathogen interactions are an outcome of co-evolution. Viruses, being obligate parasites, depend entirely on the host for their survival. The most evolved and widespread pathogens indicate better adaptability with variable hosts. Potyviruses comprise a large group of agriculturally and economically important plant viruses. The family *Potyviridae* includes the genus *Potyvirus* and nine other genera. Due to their economic and scientific importance, two potyviruses, namely potato virus Y (PVY) and plum pox virus (PPV), have been designated as being among the top ten most important plant viruses [1]. 

Most eukaryotic mRNAs have a 7-methyl guanosine (m7GpppG) cap at the 5’ end and a poly(A)-tail at the 3’ end [2]. In a cell, eIF4E/(iso)4E binds directly to the 5’cap of mRNA and functions as a component of the translation initiation complex, eIF4F/(iso)4F. The eIF4F/(iso)4F complex additionally contains the scaffold protein eIF4G/(iso)4G, the helicase eIF4A/(iso)4A, and the poly(A)-binding protein (PABP) [3,4]. This complex is responsible for mRNA 5′ cap recognition, mRNA unwinding, and the recruitment of the 40S ribosomal subunit. Instead of the cap-structure, the 5’end of the potyviral RNA is covalently linked to a protein called the viral protein genome-linked (VPg). It is a major virulence determinant of potyviruses [5,6,7,8,9,10,11]. Several investigations have confirmed that the interaction of potyviral VPg with the host eIF4E or eIF(iso)4E is necessary for potyvirus infection. This interaction has been widely studied and reviewed comprehensively in several model and crop plants including, among others, tomato, pepper, barley, lettuce, pea, bean, and mustard [12,13,14].

Potyviral VPgs may show similar affinities to both host and non-host eIFs [15]. However, a particular potyviral VPg may prefer to interact with either eIF4E, eIF(iso)4E, or both in a given host species, or they may selectively and coordinately recruit eIFs [16,17]. The preference may depend on the host. Lettuce mosaic virus (LMV) chooses eIF4E when infecting lettuce but switches to eIF(iso)4E in *Arabidopsis* [18,19]. The selection may also depend on other conditions, as exemplified by turnip mosaic virus (TuMV) VPg, which can use both the eIF4E and eIF(iso)4E of *Brassica rapa* for replication in an eIF(iso)4E knock out mutant of *Arabidopsis* [20]. The majority of the recessive resistance genes against plant viruses that have been characterized till date encode eIFs—eIF4E or its isoform eIF(iso)4E in most cases and eIF4G or eIF(iso)4G in others (reviewed in [14,21,22]). In addition, eIF2Bβ is associated with TuMV resistance in mustard plants [23]. 

Recessive resistance evolves through the deletion and point mutations in the genes that encode for host factors that are essential for the viral lifecycle [24,25]. The codons that encode the amino acids that define potyvirus resistance in eIF4E/(iso)4E and susceptibility in VPg evolve by positive selection [26]. The amino acid residues that are responsible for potyvirus resistance reside both near the cap-binding pocket of eIF4E and on the structural facet that is rotated 90° from it [27]. This may indicate that the optimal binding of VPg to eIF4E may require two binding sites, as has been suggested. The structural flexibility of VPg gives it mutational robustness, which makes VPg more prone to adaptive processes than eIF4E [28]. Many of the mutations that break eIF4E/(iso)4E-mediated resistance map to the central or C-terminal domain of VPg [8,9,10,11,24,29]. A satisfactory docking model of a PVY VPg–eIF4E complex based on VPg’s NMR structure confirms that VPg binds to the cap-binding pocket of eIF4E and competes with the m7G cap analogs that bind to that site [30]. Amino acids D111, I113, M115, Q116, L118, G119, and N121 of PVY VPg are involved in eIF4E binding, which corroborates the importance of the central domain of VPg as the binding domain. 

VPg acts as a cap-like structure to initiate viral translation. In vitro transcribed potyviral RNAs are unable to initiate infection unless they are capped, e.g., [31]. LMV VPg and the cap analogue m7GDP bind to two distinct sites of eIF4E with a similar submicromolar affinity (Kd = 0.3 µM), and the binding of either of them to eIF4E causes a 15-fold decrease in the affinity of the other [32]. When in vitro translation extracts are supplied with VPg, translation of the viral 5’ untranslated region (UTR)-containing transcripts get enhanced to some extent, whereas other mRNAs get inhibited [33,34]. A substantial eIF4E-dependent upregulation in the expression of potato virus A (PVA) RNA occurs when VPg is ectopically expressed with it in planta [35]. The quantitation of PVA gene expression was conducted in [35] by measuring virus-derived *Renilla* luciferase (3’RLUC) activity [36]. Notably, an increase in PVA RNA amounts that is comparable to the fold of increase in the 3’RLUC expression occurs upon ectopic VPg expression, thus suggesting that VPg has a role in potyviral RNA stability. Both PVA and TuMV infection induce the formation of RNA granules, which are proposed to be related to the suppression of RNA silencing [37,38]. TuMV VPg is able to resist the autophagy-mediated degradation of these granules [38], which may contribute to the stability of potyviral RNA. Recently, lettuce eIF4E was reported to be recruited by LMV particles via VPg, likely to make it immediately available for viral translation in newly infected cells [39]. In addition to the translational functions, one suggested function for the VPg–eIF4E interaction is the intracellular transportation of potyviral RNA in a complex with VPg-eIF4E-eIF4G along the microtubules [40]. The systemic spread of tobacco etch virus (TEV) is dependent on eIF(iso)4E in *Arabidopsis* [41]. The multitude of possible functions related to VPg-eIF4E binding is intriguing and calls for further studies. 

eIF4G and some other eIF4E-binding proteins carry a conserved Tyr-X-X-X-X-Leu-phi (YXXXXLΦ) recognition motif with which they bind to eIF4E [42]. In this sequence motif, X represents any amino acid, and phi is a hydrophobic amino acid. No sequence motif that resembles the consensus motif YXXXXLΦ could be found from PVY VPg structure [30]. Interestingly, such a motif, YTDIRLI, resides between amino acids 89 and 95 in PVA VPg sequence [43]. A similar eIF4E recognition motif in PVA helper-component proteinase (HCPro) has been demonstrated to bind eIF4E/(iso)4E and to be essential for PVA infection [44]. The substitution of the tyrosine and leucine residues with alanine in the eIFiso4E binding motif of HCPro has been shown to abolish binding and compromis PVA infection. A similar mutation in the PVA VPg motif reduced eIF(iso)4E binding and compromised infection [45]. 

In this study, we set out to investigate how the YTDIRLI sequence within VPg affects PVA infection and the infection-associated functions of the VPg-eIF(iso)4E interaction. We assessed the replication and post-replication functions of PVA RNA with molecules that carry mutations in the YTDIRLI encoding sequence within the VPg cistron. 

## 2. Materials and Methods 

### 2.1. Plants and Agrobacterium

*Nicotiana benthamiana* plants were grown in a greenhouse in 16 h of light at 22 °C and 8 h of dark at 18 °C. Plants were infected at the 4-to-6-leaf stage, as described previously [36]. In infiltrations, we used the *Agrobacterium tumefaciens* strain C58C1, which carried the pGV2260 helper plasmid for *vir* gene expression. 

### 2.2. Viral and Protein Expression Constructs

PVA constructs and viral protein expression constructs were based on the full-length infectious cDNA (icDNA) of PVA strain B11 (GenBank accession number AJ296311) [46]. PVA icDNA constructs for PVA^WT^, PVA^CPmut^, PVA^ΔGDD^, PVA^ΔGDDB-BOX^ and protein expression constructs for β-gluckuronidase (GUS), VPg, yellow fluorescent protein (YFP)-tagged acidic ribosomal protein P0 (P0^YFP^), eIF(iso)4E^RFP^, λN22^RFP^, were described previously [35,37,47]. The icDNA construct PVA^VPgmut^ was prepared by site-directed mutagenesis. We amplified VPg from plasmid pQKIR1-Amp-carrying 6K2-VPg fragment between SwaI and ApaI by PCR by using a pair of non-overlapping primers: VPg-SDM-Fw1 (GATGAGAGTCCCgcTACTGATATCAGGgcAATTCAGAGTC) and VPg-SDM-Rv2 (TAGTGTGTAGCCAGTTAAAGGATC). The VPg-SDM-Fw1 primer carried the point mutations in the desired sites. We PCR amplified the fragment with a high fidelity Phusion polymerase. The PCR product was treated with Dpn1 at 37 °C for 1 hr and purified. The purified PCR product was circularized with a T4 DNA ligase (Thermo scientific, Waltham, MA USA) at 4 °C overnight. The 6K2-VPg^mut^ fragment was cut out from a purified plasmid with the restriction enzymes ApaI and SwaI and inserted into pKJE23 by using the same restriction enzymes to replace the original 6K2-VPg fragment in the PVA icDNA. The mutated PVA icDNA was transferred in to a binary vector pRD400 by using KpnI and SalI restriction enzymes. In the final pRD400 construct, termed PVA^VPgmut^, PVA icDNA was under the control of the cauliflower mosaic virus (CaMV) 35S promoter and the *A. tumefaciens* nopaline synthase transcriptional terminator (*nos*).

A transient VPg^mut^ expression construct was prepared by using another set of primers, VPg-SaII-Fw (GGGGTCGACATGGGCTATAATAAGCGACAGAGGC) and VPg-KpnI-Rv (GGGGTACCCTACTCGAATTCAACCGACTCTTTC), which were used to amplify VPg^mut^ from pQKIR1-Amp. The fragment was cut by restriction enzymes KpnI and SalI and then transferred into empty pRD400 under the control of the CaMV 35S promoter and the *nos* terminator. The final expression construct was termed VPg^mut^.

### 2.3. Agrobacterium Infiltration and Sample Collection

*Agrobacterium* cells containing viral or protein expression constructs were grown in a Luria–Bertani (LB) medium at 28 °C in the presence of suitable antibiotics. The overnight cultures were sub-cultured in the same conditions for 4–5 hrs and harvested by centrifugation at 3,000 × g for 5 min. The pellets were washed once with double-distilled water, harvested again at 3,000 × g for 5 min, and then re-suspended in an induction buffer (10 mM morpholineethanesulfonic acid [MES] [pH 6.3], 10 mM MgCl_2_, and 150 μM acetosyringone). The optical density of the *Agrobacterium* suspensions was measured at 600 nm (OD_600_). The final *Agrobacterium* concentrations were adjusted by adding the induction buffer. We used *Agrobacterium* carrying PVA constructs with an OD_600_ of 0.05 and protein expression constructs with an OD_600_ of 0.3 unless otherwise stated. We used a firefly luciferase (FLUC) expression construct as an internal control for normalization of 3’RLUC activities at OD_600_ of 0.005. *Agrobacterium*-carrying virus and protein constructs were infiltrated alone or in combinations at the required ratios in the final infiltration mix. The infiltration mixes were incubated for 2 to 3 h in the induction buffer at room temperature prior to infiltration. Infiltration was performed with a syringe to the abaxial side of *N. benthamiana* leaves. Sampling was done by cutting 5-to-10-mm leaf discs with a cork borer surrounding the infiltrated region at different time points, as explained in the results. Each sample contained four leaf discs pooled from 3–4 infection foci. The collected samples were immediately frozen in liquid nitrogen and then either stored at −80 °C or used immediately. Each experiment was performed at least twice with a minimum of three biological replicates.

### 2.4. Quantification of Viral Gene Expression by 3’RLUC Assay

We quantitated viral gene expression by performing a 3’RLUC assay, as described previously [36]. The 3’RLUC expression was measured by a Luminoscan TL Plus instrument (Thermo Lab systems) by using a dual luciferase kit (Promega, Madison, Wisconsin, United States). Infected leaf samples were prepared according to the manufacturer’s instructions. 3’RLUC normalization was done with the following formula: normalized 3’RLUC activity = (average FLUC activity/FLUC activity per sample) × 3’RLUC activity per sample. Average normalized 3’RLUC values and their standard deviations (SD) were calculated from each sample set. A Student’s *t*-test was performed to calculate the significance of the differences between the experimental and control samples.

### 2.5. Reverse Transcription-Quantitative PCR

We performed reverse transcription-quantitative PCR (qRT-PCR), from the infected *N. benthamiana* leaf samples in three different types of experiments. Firstly, the total viral RNA was quantitated. The second type of qRT-PCR was specific for the negative-strand of the PVA RNA, and the third one was done to quantitate the RNA associated with coat protein (CP) by immune capture qRT-PCR (IC-qRT-PCR). 

Infected leaf discs were collected at different time point, as mentioned in the results. Each sample set represented a pooled sample from four infection foci. The leaf samples were immediately frozen and grounded in liquid nitrogen. RNA isolation was carried out with an RNeasy plant minikit (Qiagen, Hilden, Germany) according to the manufacturer’s protocol. One microgram of total RNA from each sample was treated with RNase-free DNase (Thermo Scientific). First-strand cDNA synthesis was performed by using a RevertAid H Minus first-strand cDNA synthesis kit (Thermo Scientific) according to the user protocol. For the quantitation of the total RNA amount, the random hexamers provided with the kit were used to prime the cDNA synthesis. For the negative-strand RNA quantification, a minus strand-specific forward primer (CPqPCRF’) was used for the cDNA synthesis. For IC-qRT-PCR, no RNA isolation was performed before cDNA synthesis; instead, CP assemblies and/or intact PVA particles were captured from the infected leaf sample. The grounded samples for this experiment were mixed with 200 µL of a sample extraction buffer (wash buffer, 8 M polyvinylpyrrolidone [PVP], and 0.2% BSA). The samples were allowed to settle on ice for 30–45 min. Ninety-six-well plates that were coated with anti PVA CP antibodies were used to capture the CP assemblies from the infected leaf samples. For coating, the PVA antibody (PVA mix MAb; Science and Advice for Scottish Agriculture [SASA], Edinburgh, UK) was diluted to 1:1000 in the coating buffer (15 mM Na_2_CO_3_ and 34 mM NaHCO_3_ [pH 9.6]) and incubated in the wells for 3 h at 37 °C. Plates were washed 3 to 4 times with a wash buffer (1.4 mM KH_2_PO_4_, 8 mM Na_2_HPO_4_, 136 mM NaCl, 2.6 mM KCl [pH 7.4], and 0.05% Tween 20) to remove the excess unbound antibody, and then 100 µL of leaf samples were added in each well of the coated plate before being incubated overnight at 4 °C. A non-infected plant sample extract was used as a negative control for the experiment. Plates were gently washed with thewash buffer and were then used for cDNA preparation. qRT-PCR was then performed in 96-well plates by using a CFX96 Touch real-time PCR system (Bio-Rad, Berkeley, California). Each reaction mixture of 10 μL in volume contained 5 μL of Maxima SYBR green qPCR master mix (Thermo Scientific), 0.5 μM of each forward and reverse primers, 1 μL of cDNA, and 3 μL of nuclease-free water. Three technical replicates were performed from each cDNA sample. The following primers were used: CPqPCRF′ (5′-CATGCCCAGGTATGGTCTTC-3′) and CPqPCRR′ (5′-ATCGGAGTGGTTGCAGTGAT-3′). The housekeeping gene protein phosphatase 2A (PP2A) was used as a reference gene. PP2A was amplified with the primers, as described previously [48]. The amplification parameters for qPCR were 3 min of initial denaturation at 95 °C, followed by 39 cycles of 10 s of denaturation at 95 °C, 30 s of annealing 55 °C, and 30 s of synthesis at 72 °C. A melting curve was generated by heating from 60 to 95 °C in increments of 0.5 °C/s. The following controls were included: a template replaced by nuclease-free distilled water (dH_2_O) as a non-template control and RT reaction mixtures that lacked the reverse transcriptase as non-RT controls. A serial dilution of PVA icDNA was used, and the quantification cycle (Cq) values were plotted against the values for the input cDNA to construct the standard curve.

### 2.6. Confocal Microscopy

In the co-localization study of λN22^RFP^ or eIF(iso)4E^RFP^ with P0^YFP^, the living tissues of the infected *N. benthamiana* leaves were examined at 3 days post infiltration (dpi). Four leaf discs surrounding the infiltration point were cut with a cork borer and mounted in a slide with a few drops of water. Leaf discs were placed under cover glass, and the abaxial side of the leaf discs was scrutinized under confocal laser scanning microscopy, Leica True Confocal Scanning-Spectral Photometric 5 (Leica TCS SP5II, Wetzlar, Germany). For visualizing YFP, excitation was performed with an argon laser at 488 nm, and emission was recorded at 525–555 nm. RFP was excited with Diode Pumped Solid State (DPSS) 561 nm laser and emission recorded at 570–620 nm (DD 488/561 beam splitter). The sequential scanning mode was applied for the co-imaging of fluorescent proteins. Single cells were magnified under a 63× water immersion objective to acquire images. All images presented in the figures are acquired at the same magnification. The co-localization analysis of P0^YFP^ with λN22^RFP^ or eIF(iso)4E^RFP^ were performed with the Fiji (ImageJ; www.imagej.net) image analysis software package by using the co-localization threshold-function. P0^YFP^-containing granules were selected as regions of interest for quantitation of co-localization, and the results are given as the % intensity of P0^YFP^ co-localizing with λN22^RFP^ or eIF(iso)4E^RFP^. A bar diagram was made to indicate the percentages of co-localization and a Student’s *t*-test was employed to calculate the significance of the differences between the experimental and control samples.

### 2.7. Quantification of PGs by Epifluorescence Microscopy

PGs were quantified at 3 dpi from infected *N*. *benthamiana* leaves by using epifluorescence microscopy (Zeiss Axio Scope.A1 microscope, Jena, Germany). Four leaf discs, surrounding the infiltration point were cut out from each plant. Leaf discs were mounted in a glass slide with a few drops of water and placed under a cover glass. An appropriate filter for YFP was used to visualize PGs. PGs were counted in the epidermal tissue under 20× magnification. The average number of PGs found in 1 mm^2^ view area from each set is presented in a bar diagram. For each condition, three different plants were used, and from each plant, PGs were counted from four separate leaf areas (*n* = 12). Standard deviation was calculated. 

### 2.8. Electron Microscopy (EM)

To observe the PVA particles, infected leaf samples were collected at 7 dpi from local leaves. Four leaf discs of approximately 100 mg of leaf tissue was ground in liquid nitrogen and mixed with a 0.06 M phosphate buffer. Leaf samples were incubated in ice for about 1 h to let the cell debris settle. Carbon-coated electron microscopy (EM)-grids were incubated with the anti-CP antibodies (PVA mix MAb; Science and Advice for Scottish Agriculture [SASA], Edinburgh, UK) that were diluted to 1:100 for 1 h at room temperature. The excess antibody was washed with phosphate buffer. Antibody-coated grids were incubated at 4 °C overnight with supernatant that was collected from the leaf extracts. Grids were further washed with 20 drops of a phosphate buffer and immediately stained with 2% uranyl acetate for 15 seconds. Excess stain was drenched from the grids by using filter paper, and dried grids were used for the visualization of the particles with a Jeol JEM-1400 transmission electron microscope (Jeol Ltd., Tokyo, Japan). 

## 3. Results

### 3.1. The Central Region of PVA VPg Carries a Consensus Sequence for eIF(iso)4E Binding

**Figure 1:**
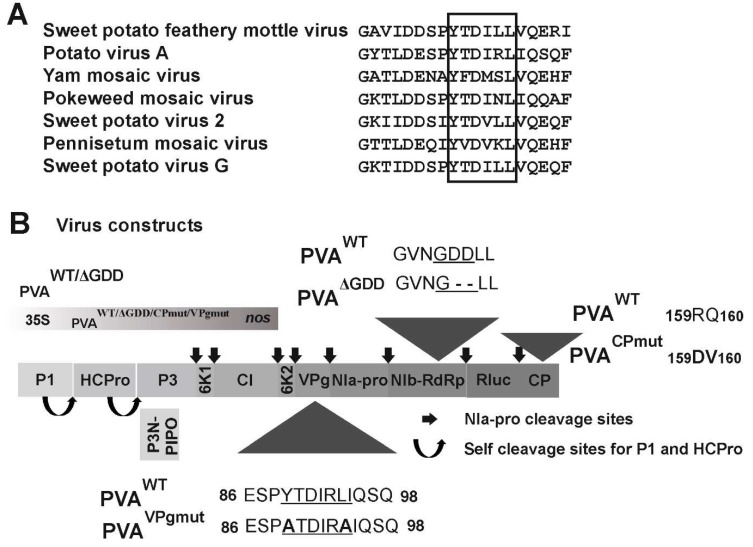
eIF4E-binding motif Tyr-X-X-X-X-Leu-phi (YXXXXLΦ) within potato virus A (PVA) viral protein genome-linked (VPg). (**A**) Alignment of the YXXXXLΦ motives in potyviruses. Out of the 112 studied potyviruses, an eIF4E-binding motif YXXXXLΦ could be found only from the VPg sequence of the indicated seven viruses. (**B**) A schematic presentation of full-length PVA cDNA constructs used in this work. Mutations in the YXXXXLΦ motif in PVA^VPgmut^ substitute the conserved tyrosine and leucine residues of PVA VPg with alanine. The deletion of the aspartate residues of the GDD motif in PVA^ΔGDD^ nuclear inclusion protein b (NIb) results in a replication-deficient version of PVA. The substitution of amino acids RQ in the middle of the coat protein (CP) with DV in PVA^CPmut^ results in a movement-deficient version of PVA.

The alignment of 112 potyviral VPgs revealed that the putative eIF4E binding site YXXXLΦ, which can be found in the central region of PVA VPg (YTDIRLI, amino acids 89–95 of PVA VPg), is not conserved throughout potyviruses. VPgs of only seven potyviruses carry this motif (Figure 1A). In this study, we set to investigate the role of the YTDIRLI, the consensus sequence for eIF4E binding [49], in the functions of VPg during PVA infection. We established a method for the sensitive quantitation of gene expression from PVA RNA. It is based on an infectious cDNA (icDNA) clone of PVA (PVA^WT^) that expresses 3’RLUC as part of the viral polyprotein from a cistron between nuclear inclusion protein b (NIb; RNA-dependent RNA polymerase) and CP cistrons in PVA RNA [36]. To enable the quantitation of PVA gene expression in the absence of the consensus eIF(iso)4E-binding motif YXXXLΦ, we replaced the tyrosine and leucine codons of the YTDIRLI encoding sequence in PVA icDNA with those encoding for alanine, similarly to that shown in [45]. This construct was named PVA^VPgmut^ (Figure 1B). In order to understand how PVA^VPgmut^ RNA functions in planta, we used two other PVA RNA mutants for comparison: one that is not able to replicate, called PVA^ΔGDD^, and one that replicates but is confined to the primarily transformed cells due to a cell-to-cell movement defect called PVA^CPmut^ (see Figure 1B for details) [36].

### 3.2. Low Level of Viral Replication Occurs in N. benthamiana Leaves Infiltrated with PVA^VPgmut^

**Figure 2:**
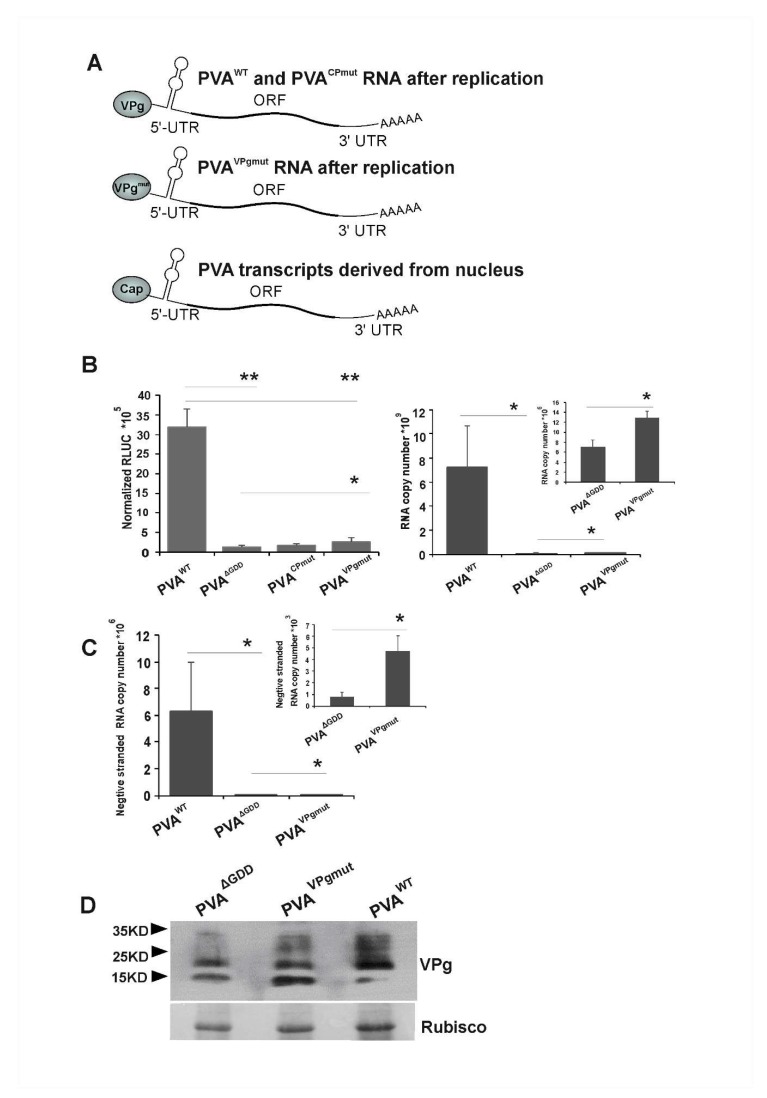
Viral replication occurs in PVA^VPgmut^-expressing *Nicotiana benthamiana* cells. (**A**) A schematic representation of PVA RNA prior to replication and after the first round of replication in *Agrobacterium*-infiltrated *N. benthamiana* leaves. Importantly, PVA RNAs appear from the nucleus as capped transcripts and only after the first round of replication carry VPg at their 5’end. (**B**,**C**) PVA^WT^, PVA^ΔGDD^, PVA^CPmut^ and PVA^VPgmut^ cDNAs were *Agrobacterium*-infiltrated into *N. benthamiana* leaves at OD_600_ 0.05. In (**B**), samples were collected at 3 dpi followed by a luciferase assay to determine virus-derived *Renilla* luciferase (3’RLUC) activity (left panel; (*n* = 4)) and qRT-PCR to quantitate viral RNA accumulation (right panel; (*n* = 4); PVA^CPmut^ RNA accumulation was not determined). In (**C**), samples were collected at 4 dpi followed by the quantitation of PVA (-)-strand RNA accumulation (*n* = 4). The significance of the differences between the constructs in representative experiments was calculated by a Student’s *t*-test with pair-vice comparisons, as indicated in the figure. *p*-values < 0.01 are indicated with ** and < 0.05 with *. (**D**) Samples collected from *N. benthamiana* leaves that were infiltrated with *Agrobacterium* carrying PVA^WT^, PVA^ΔGDD^ and PVA^VPgmut^ cDNAs (OD_600_ 0.05) at 3 dpi were subjected to a Western blot analysis with anti-VPg antibodies to verify VPg/VPg^mut^ accumulation.

Next, we quantitated viral gene expression from PVA^VPgmut^ RNA with the aid of 3’RLUC activity. Our question here was whether PVA^VPgmut^ gene expression resembled that of the replicating or non-replicating PVA RNA. The experiment was initiated by infiltrating *Agrobacterium* carrying PVA^VPgmut^ construct (OD_600_ 0.05) into *Nicotiana benthamiana* leaves. For comparison, we infiltrated similarly *Agrobacterium* carrying PVA^WT^, PVA^ΔGDD^ and PVA^CPmut^ constructs. 3’RLUC activity coming from the non-replicating PVA^ΔGDD^ RNA was translated from capped viral transcripts that were derived from nucleus, as illustrated in Figure 2A. This was also the case for the PVA^WT^, PVA^CPmut^ and PVA^VPgmut^ RNAs emerging from the nucleus prior to the first round of replication. We assumed that the replicated PVA^CPmut^ and PVA^WT^ RNAs carried wild type VPg at their 5’ends. This assumption was based on studies that have shown that PVA VPg gets uridylylated by the catalytic activity of NIb and may therefore serve as an uridylylated protein primer for replication [50]. PVA RNA that is encapsidated to PVA particles carries VPg at its 5’end [51]. Potyviral RNA is encapsidated only if it is able to replicate [52,53]. If PVA^VPgmut^ replicates, according to the same logic, PVA^VPgmut^ RNA ought to carry VPg^mut^ protein at its 5’end, as schematically drawn in Figure 2A. The 3’RLUC activities reporting for the viral gene expression from PVA^VPgmut^, and the other PVA RNAs were determined at 3 dpi (Figure 2B). In line with our previous results, we found that PVA^ΔGDD^ gene expression was lower than that of PVA^WT^ and PVA^CPmut^. PVA^VPgmut^ 3’RLUC activity was higher than that of PVA^ΔGDD^ and PVA^CPmut^, but it was significantly lower than that of PVA^WT^. These 3’RLUC activity levels suggested that PVA^VPgmut^ RNA, or at least the capped PVA^VPgmut^ transcripts derived from the nucleus, may replicate. 

As the 3’RLUC expression level did not provide an ultimate proof for PVA^VPgmut^ RNA replication, more data were needed. Next, we infiltrated *Nicotiana benthamiana* plants with PVA^VPgmut^, PVA^WT^ and PVA^ΔGDD^ for the detection of the viral minus-strand (−)RNA. PVA^WT^ and PVA^ΔGDD^ were used as a positive and a negative control, respectively. We collected the infected leaf samples at 4 dpi and quantitated PVA (−)RNA by qRT-PCR. In three parallel experiments, PVA^VPgmut^ samples contained an approximately fivefold higher amount of (−)RNA than PVA^ΔGDD^, though this was still 1000 fold less than PVA^WT^ (Figure 2C). These data further supported the possibility that PVA RNA replicates in PVA^VPgmut^-expressing plants. As this may consequently lead to reversion to PVA^WT^, it is not clear whether the increase in the (+)-strand RNA in Figure 2B and (−)-strand RNA in Figure 2C in comparison to PVA^ΔGDD^ came from PVA^VPgmut^ RNA or from its reversion to PVA^WT^. 

A Western blot analysis with anti-VPg antibodies of samples that were collected at 3 dpi from infiltrated *Nicotiana benthamiana* leaves showed an accumulation of VPgs from PVA^WT^ and PVA^ΔGDD^, as well as VPg^mut^ from PVA^VPgmut^ (Figure 2D). There is a relatively small difference in the VPg and VPg^mut^ amounts that were expressed from these constructs if compared to the corresponding RNA amounts (see Figure 2B, right panel). Accordingly, in our previous study, the difference in the amounts of viral cylindrical inclusion (CI) protein, whether expressed from a replicating or a non-replicating PVA RNA in planta, was small and did not correlate with the viral RNA amounts [54]. These results showed that a substantial amount of VPg and VPg^mut^ was present in the infiltrated leaves of the PVA^ΔGDD^ and PVA^VPgmut^-expressing plants, respectively. The results also suggested that a major part of PVA^WT^ RNA was encapsidated and not available for gene expression. 

### 3.3. PVA^VPgmut^ Fails to Move Systemically but Reverts Readily to PVA^WT^

**Figure 3:**
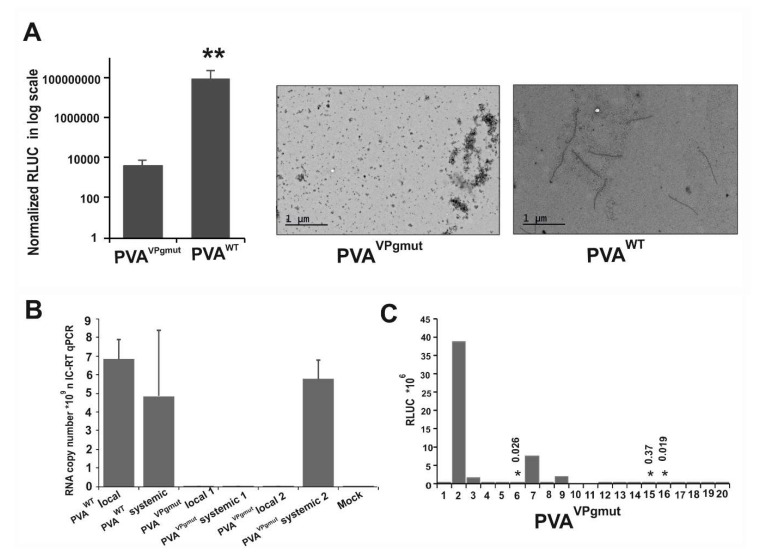
PVA^VPgmut^ genome reverts to PVA^WT^. PVA^WT^ and PVA^VPgmut^ cDNAs were *Agrobacterium-*infiltrated into *N. benthamiana* leaves at an OD_600_ of 0.05. In (**A**) the samples were collected at 7 dpi followed by a RLUC assay to determine virus-derived 3’RLUC activity (left panel; (*n* = 6)). A Student’s *t*-test showed the significance of the difference between PVA^VPgmut^ and PVA^WT^; *p*-value < 0.01 is indicated with **. Particle formation in the infiltrated leaves at 7 dpi was investigated by electron microscopy. No particles were detected in PVA^VPgmut^-containing leaves. (**B**) The accumulation of CP-associated viral RNA was quantitated in the local and systemic leaves of *N. benthamiana* 10 dpi by IC-qRT-PCR. RNA copy numbers in the local and systemic leaves of PVA^VPgmut^-infiltrated plants were determined from two individual plants (indicated as 1 and 2). (**C**) 3’RLUC expression in the systemically-infected *N. benthamiana* leaves following the reversion of PVA^VPgmut^ to PVA^WT^. As verified by sequencing, reversion occurred in this experiment in seven out of 20 plants. The 3’RLUC values that are too low to see as a distinguishable column are marked by asterisks, and the corresponding 3’RLUC value is given.

We reasoned that if the PVA^VPgmut^ was able to replicate even on a low level, there was a possibility that the mutant virus would revert to PVA^WT^. We *Agrobacterium*-infiltrated *Nicotiana benthamiana* plants with PVA^VPgmut^ and PVA^WT^, and we collected leaf samples from the infiltrated leaves at 7 and 10 dpi. In the light of the current knowledge, the formation of virus particles is a strong proof for potyvirus replication [53]. However, in contrast to PVA^WT^, we could not detect any particles from the local leaves of PVA^VPgmut^-expressing plants by electron microscopy at 7 dpi (Figure 3A). This was also essentially the case when the amount of particles was quantitated by immuno-capture (IC)-qRT-PCR from the local leaves at 10 dpi (Figure 3B). Numbers 1 and 2 in Figure 3B indicate the IC-qRT-PCR results presented from two individual plants that expressed PVA^VPgmut^ RNA. The amount of PVA^VPgmut^ RNA associated with particles was close to the background levels of the non-infected mock sample and far below the PVA^WT^ RNA amounts (Figure 3B). When the samples were collected from the systemic leaves of these same plants at 10 dpi, we detected a comparable amount of particle-associated PVA RNA in PVA^VPgmut^-infiltrated plant number 2, as in PVA^WT^-infiltrated plant (see Figure 3B). We suspected that a reversion to PVA^WT^ had occurred in this plant and therefore decided to follow the possible development of systemic infection in a large amount of PVA^VPgmut^-infiltrated plants. We kept PVA^VPgmut^-infiltrated plants in a separate growth chamber to avoid any contamination from PVA^WT^-infected plants. Samples for the detection of systemic infection were collected depending on the experiment at 10, 13, or 15 dpi. We found that when all these experiments were combined, 26% of PVA^VPgmut^-infected plants (13 out of 50 plants) expressed virus-derived 3’RLUC in the systemic leaves. The 3’RLUC activities that were determined in systemic leaves at 10 dpi in a representative experiment are given in Figure 3C. We isolated the total RNA from the systemically-infected leaves, synthesized cDNA, and sent the samples for sequencing. In all of the samples that expressed virus-derived 3’RLUC, it came from PVA^WT^ RNA. Thus, the data indicated that systemic infection and particle formation in PVA^VPgmut^-infiltrated plants can only occur after reversion to PVA^WT^. We assumed that reversion occurred only in few cells within the leaves infiltrated with PVA^VPgmut^, and, therefore, particle formation remained hard to detect from the local leaves. 

### 3.4. VPg^mut^ is Not Able to Enhance PVA^WT^ RNA Stability and 3’RLUC Expression

**Figure 4:**
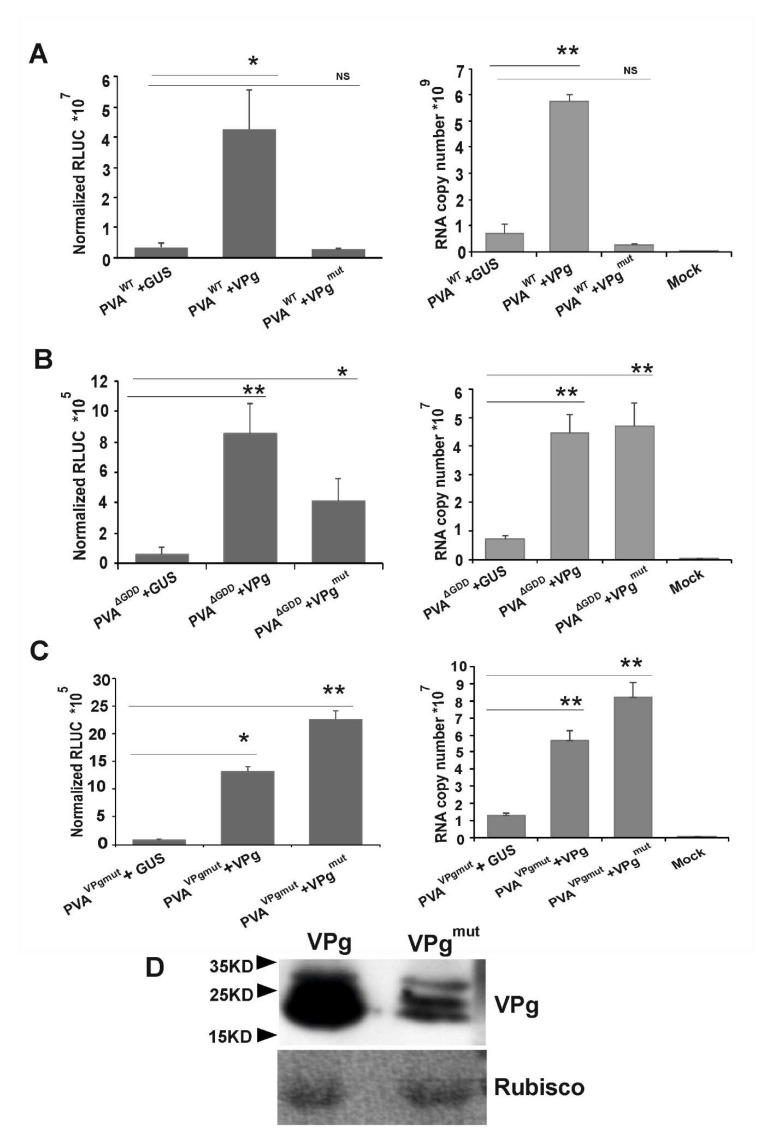
eIF4E-bidning motif YXXXXLΦ is required in the in trans expressed VPg for enhanced PVA^WT^ 3’RLUC expression and RNA stabilization. Normalized 3’RLUC activity and RNA accumulation from (**A**) PVA^WT^, (**B**) PVA^ΔGDD^ and (**C**) PVA^VPgmut^ was determined in the presence of ectopically expressed GUS, VPg and VPg^mut^ at 3 dpi. Experiments were performed at least in triplicate. Data represent one representative experiment, and the bars display mean ± S.D (*n* = 3 or more). A Student’s t-test shows which of the samples were significantly different compared to the GUS control; *p*-values < 0.05 are indicated with *, and *p-*values < 0.01 are indicated with **. (**D**) Samples collected from *N. benthamiana* leaves that were infiltrated with *Agrobacterium* carrying VPg and VPg^mut^ expression constructs (OD_600_ 0.5) at 3 dpi were subjected to a Western blot analysis with anti-VPg antibodies to verify VPg/VPg^mut^ accumulation.

Previously, we have shown that ectopically expressed VPg enhances both PVA^WT^ and PVA^ΔGDD^ RNA accumulation and gene expression [35,37,47]. Recently, we published an observation that virus-derived RLUC expression is enhanced only if the *RLUC* gene is located between NIb and CP encoding cistrons in PVA RNA and not if it resides in front of the HCPro encoding cistron [54]. Here, we wanted to understand how VPg^mut^ behaves in respect to the enhancement of viral RNA accumulation and 3’RLUC expression. We co-infiltrated PVA^WT^, PVA^ΔGDD^ and PVA^VPgmut^ and either VPg, VPg^mut^ or GUS with *Agrobacterium*. GUS expression with PVA RNAs served here as the baseline. PVA RNA accumulation and 3’RLUC activity were both quantitated from the samples that were collected from the infiltrated local leaves at 3 dpi. In line with our previous results [35,54], in trans expressed VPg boosted the accumulation of 3’RLUC activity by approximately 15 fold and boosted PVA^WT^ RNA accumulation by approximately 10 fold (Figure 4A). Interestingly, this did not happen when VPg^mut^ was co-expressed together with PVA^WT^ (Figure 4A). In contrast, both VPg and VPg^mut^ enhanced the RNA accumulation and 3’ RLUC expression of PVA^ΔGDD^ (Figure 4B) and PVA^VPgmut^ (Figure 4C). As PVA^ΔGDD^ does not replicate, free VPg/VPg^mut^ contributed here to the stability of the viral RNA and not to the capacity to replicate. The ectopically expressed VPg could not complement the debilitated PVA^VPgmut^ infection to the level of the PVA^WT^ infection (see Figure 4C), since the PVA^VPgmut^ RNA copy number (>10^7^) remained two magnitudes of order lower than the PVA^WT^ RNA copy number (>10^9^). The accumulation of VPg and VPg^mut^ from the corresponding expression vectors was verified by Western blotting (Figure 4D). 

Taken together, these results propose that after replication, newly synthesized PVA^WT^ RNA needs free cytoplasmic VPg for its stabilization and 3’RLUC expression. The results further point out to the possibility that both genome-linked and free VPgs need to be capable to interact with eIF4E in order to stabilize PVA RNA and establish infection.

### 3.5. The Amount of PVA-Induced Granules Increases in the Presence of VPg^mut^

Silencing suppression is one of the major strategies to protect viral RNA from degradation. One feasible possibility as to why PVA^VPgmut^ is not able to establish infection is that the PVA^VPgmut^ RNA ends up degrading after replication, as suggested by its low level (see Figure 2B, right panel). We have published that HCPro induces granules during PVA-infection, and we have proposed that they are a consequence of the RNA silencing suppression function of HCPro [37]. We call these granules potyvirus-induced granules (PGs). They contain PVA RNA, HCPro, eIF(iso)4E, and many other host RNA-binding proteins [37]. One possibility behind the inability of PVA^VPgmut^ to establish infection could be that the VPg-eIF(iso)4E interaction is required together with HCPro to assist the replicated VPg-linked PVA RNA to silencing suppression pathway and translation. With this hypothesis in mind, we wanted to analyze how PGs behaved in the presence of PVA^VPgmut^ and whether free VPg^mut^ had any effect in that. 

**Figure 5:**
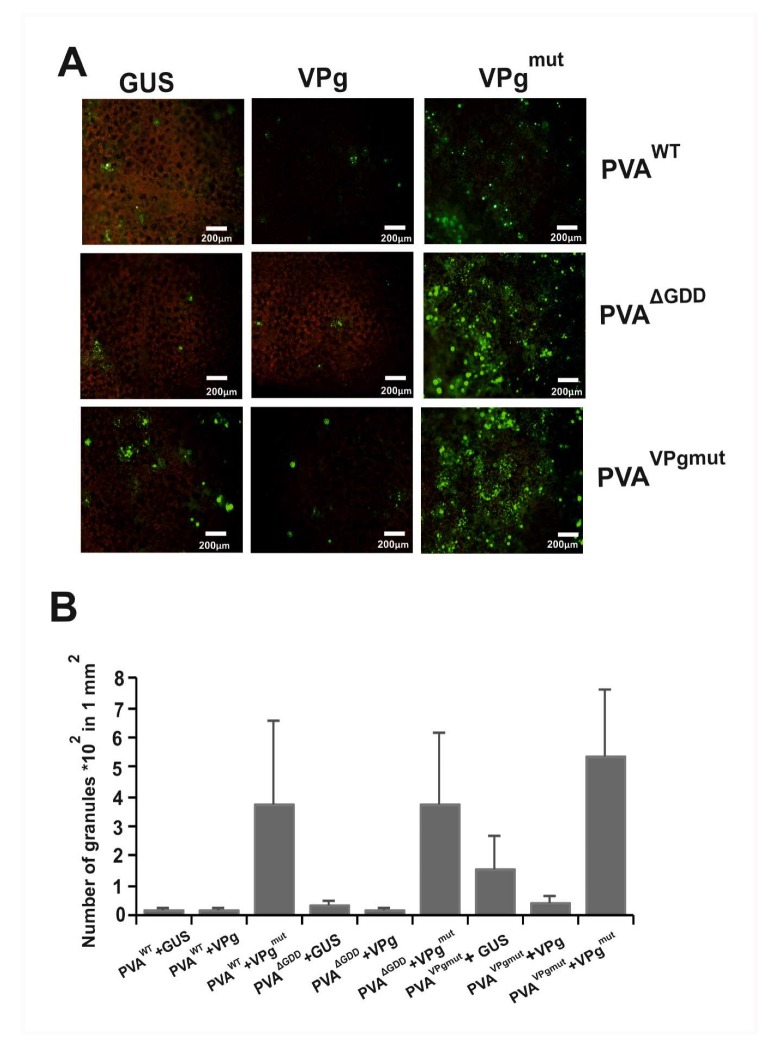
The number of P0-containing PGs increases in the presence of VPg^mut^. (**A**) P0^YFP^ (OD_600_ 0.3) and either PVA^WT^, PVA^ΔGDD^, or PVA^VPgmut^ (OD_600_ 0.05) were *Agrobacterium-*infiltrated in *N. benthamiana* leaves to induce PGs, and the effect of co-expressed GUS, VPg and VPg^mut^ on P0^YFP^-labeled PGs was examined by epifluorescent microscopy three days later. Scale bar: 200 µm. (**B**) The number of P0^YFP^-labeled PGs/mm^2^ was calculated in leaf tissues during the expression of either GUS, VPg or VPg^mut^.

We infiltrated *Agrobacterium*carrying PVA^WT^, PVA^ΔGDD^, and PVA^VPgmut^ constructs along with the expression constructs for GUS, VPg, and VPg^mut^ in different combinations (Figure 4A). We used YFP-tagged acidic ribosomal protein P0 (P0^YFP^), which co-localizes together with HCPro in PGs [37], as a marker protein for the detection of PGs in fluorescence microscopy. *Agrobacterium* containing P0^YFP^ expression construct was infiltrated together with each combination of the other constructs. We calculated the number of foci that contained P0^YFP^ in a defined area of an infiltrated leaf under epifluorescence microscopy at 3 dpi (Figure 5). We found that the number of PGs in plants that expressed PVA^WT^ and PVA^ΔGDD^ together with either GUS or VPg was lower than that in the presence of in trans expressed VPg^mut^. When co-expressed together with GUS, PVA^VPgmut^ induced more P0-containing foci than either PVA^WT^ or PVA^ΔGDD^. Their number further increased when PVA^VPgmut^ was co-expressed with VPg^mut^ and reduced when expressed with VPg. These results implied that the presence of VPg^mut^ boosted the production of P0-containing foci. To understand the nature of these abundant foci, we went on to check for the presence of viral RNA and eIFiso4E, which have both been shown to be components of PGs [37]. 

### 3.6. The Amount of Viral RNA is Reduced in P0-Contining Foci in the Presence of VPg^mut^

**Figure 6:**
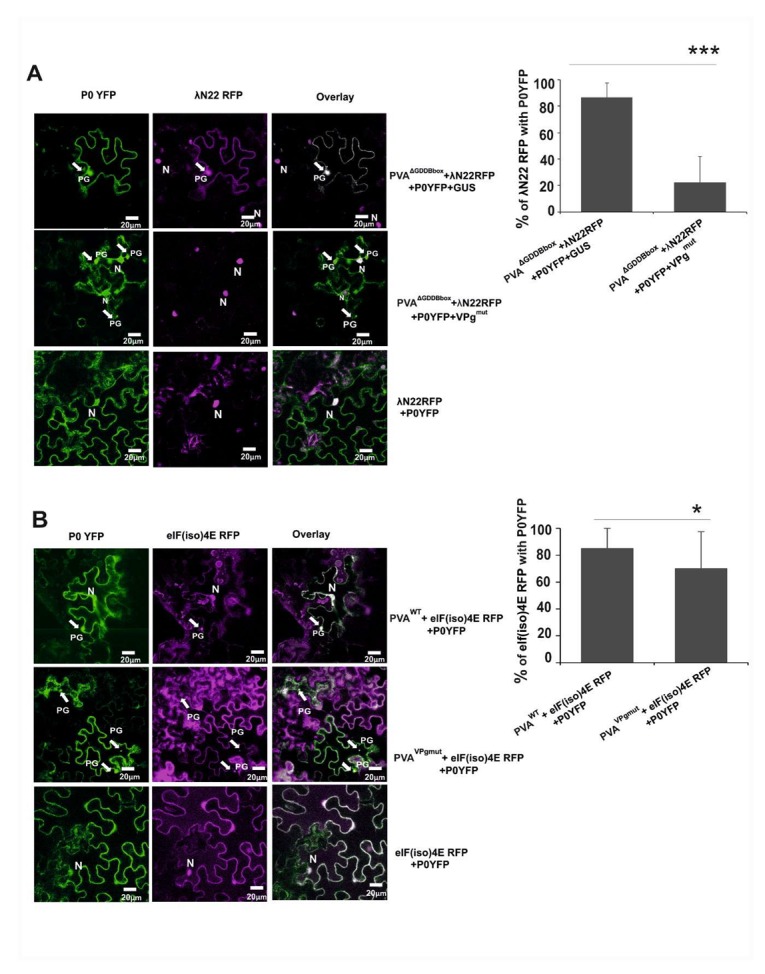
PGs formed in the presence of VPg^mut^ contained reduced amounts of PVA RNA. (**A**) The binding of λN22^RFP^ to the bacteriophage λ B-box RNA elements within the 3´UTR of PVA^∆GDD^ RNA enabled the visualization of PVA RNA in vivo. PGs were induced by PVA^ΔGDDB-box^ and visualized by P0^YFP^ (green channel) and λN22^RFP^ (magenta channel). The RFP signal was mainly found in the nucleus (indicated by N) due to the nuclear localization signal that was present in λN22^RFP^, but it was also found in the cytoplasm and PGs if bound to PVA^ΔGDDB-box^ RNA. The images are projections of Z-stacks with PGs indicated by arrows. Scale bar: 20 µm. The percentage of P0 and PVA^ΔGDDB-box^ RNA co-localization in PGs (*n* = 22) was quantitated with the Image J program. (**B**) PGs were induced either by PVA^WT^ or PVA^VPgmut^ and visualized by P0^YFP^ (green channel) and eIF(iso)4E^RFP^ (magenta channel). Substantial co-localization between the YFP and RFP signals was observed. The images are projections of Z-stacks with PGs indicated by arrows. Scale bar: 20 µm. The percentage of P0 and eIF(iso)4E co-localization in PGs that were induced by PVA^WT^ and PVA^VPgmut^ (*n* = 47) was quantitated with the Image J program. A Student’s *t*-test shows the difference between the samples; *p*-values <0.05 are indicated with *, and *p-*values < 0.001 are indicated with ***.

We were interested in checking the components within PGs in the presence of VPg^mut^. Our main interest was to see whether those PGs contained PVA RNA. Previously in [37], we showed that PGs contain PVA^ΔGDD^ RNA. We used λN22 and a B-box element-based detection system to detect PVA RNA in PGs. λN22 protein has an affinity to B-box RNA element of lambda phage [55]. The cloning of sixteen B-box elements to the end of PVA 3’UTR in a PVA^ΔGDD^ construct (PVA^ΔGDD B-box^) and an RFP gene to a λN22 encoding sequence in the λN22^RFP^ construct were described in [37]. We *Agrobacterium-*infiltrated *Nicotiana benthamiana* plants with a PVA^ΔGDD B-box^ construct along with the λN22^RFP^ and P0^YFP^ constructs and with either a GUS or VPg^mut^ construct. The co-localization of the λN22^RFP^ and P0^YFP^ signals in the PVA^ΔGDD B-box^-infiltrated and GUS-expressing samples verified the existence of viral RNA in PGs (Figure 6A; upper panels). The majority of the granules produced in the leaf samples that expressed VPg^mut^ did not co-localize with the λN22^RFP^-signal, which reports for the localization of PVA^ΔGDD B-box^ RNA (Figure 6A; middle panels). Out of the 76 granules studied by eye, we detected five granules, which contained PVA^ΔGDD B-box^ RNA. The quantitation of 22 randomly selected granules by the ImageJ program resulted in an overall percentage of co-localization around 20%, whereas the corresponding percentage for PVA^ΔGDD B-box^ RNA in the absence of VPg^mut^ was 86%. 

As this study was about a mutation in the general eIF4E consensus binding site, the next logical step was to check if eIF(iso)4E, which is a component of PGs [37], is present in the P0-containing foci in cells that express VPg^mut^. eIF(iso)4E tagged with RFP was expressed together with P0^YFP^ and either PVA^WT^ or PVA^VPgmut^ in *N. benthamiana* leaves. The eIF(iso)4E^RFP^-signal co-localized with P0^YFP^ in granules within PVA^WT^-infected cells (Figure 6B; upper panels) at a rate of more than 86% (*n* = 47). In the presence of PVA^VPgmut^, the percentage of the co-localization of P0 and eIF(iso)4E was reduced to 70% (Figure 6B; lower panels). As a negative control, we infiltrated *Nicotiana benthamiana* plants with P0^YFP^ and λN22^RFP^ without a virus. λN22 has a nuclear localization signal and is only retained in the cytoplasm upon binding to cytoplasmic B-box labelled RNAs [55]. No PG-like structures were formed in the negative control sample. λN22^RFP^ was found from nucleus, and P0^YFP^ expression could be detected from all over the cytoplasm and occasionally from the nucleus (Figure 6A; lower panels). This indicates that in the presence of VPg^mut^, these PGs were not similar to PGs described in [37], as these foci contained less PVA RNA. 

## 4. Discussion

The interaction between VPg and eIF4E/(iso)4E is among the most important factors supporting potyvirus infection [24]. However, the exact nature of the molecular functions of the VPg-eIF4E interaction in potyvirus infection has remained elusive. In this study, we approached the functions of the VPg-eIF(iso)4E interaction in PVA infection by dissecting the infection process with the mutated PVA^VPgmut^ that carried similar mutations in the conserved eIF4E binding site YXXXXLΦ in PVA VPg as in [43,45]. In [45], the authors demonstrated that this binding site contributes to the capacity of PVA VPg to bind eIF(iso)4E and reported that it is crucial for the infectivity of PVA. They also demonstrated that nuclear and nucleolar localization of VPg is not altered by the mutations. Our results suggest that the capacity of PVA VPg to interact with eIF(iso)4E via this site is dispensable for replication but virtually indispensable for passing the replicated RNA through host’s antiviral defense to translation. 

In our study system, following *Agrobacterium* infiltration, T-DNAs that carried PVA cDNAs were transferred to the host cell nucleus, where the capped PVA transcripts entered cytoplasm and the first round of viral translation. The capped PVA^VPgmut^ transcripts produced VPg^mut^ before entering the replication complex. Wild type VPg can be available in PVA^VPgmut^-expressing cells only if a reversion to PVA^WT^ RNA occurs. The accumulation of the (−)-strand PVA^VPgmut^ RNA suggests that the capped transcript could serve as a template for replication. In accordance, it has been proven that in vitro transcribed capped full-length PVA RNAs replicate and consequently initiate infection, e.g., [31]. Accumulation of (-)-strand also proposes that VPg^mut^ can become uridylylated similarly to the wild type potyviral VPgs [50,56] and serve as an uridylylated protein primer for replication similarly to picornaviruses [57]. That is why we see it as possible that, after the first round of replication, PVA^VPgmut^ RNA carries VPg^mut^ at its 5’end (see Figure 2A). To obtain a final proof for this assumption would require the isolation of PVA RNA carrying VPg^mut^ at its 5’end, followed by mass spectrometry for the identification of the peptide that carried the mutation. This is technically demanding due to the low accumulation of PVA^VPgmut^ RNA (see Figure 2B). 

Engineered mutations in viral RNA that are disadvantageous for infection tend to revert to wild type RNA during replication. We observed a reversion of PVA^VPgmut^ RNA to PVA^WT^ RNA in 26% of our experimental plants. In the cells in which reversion happened, the replication of capped PVA^VPgmut^ RNA purportedly resulted first in the formation of PVA^WT^ RNA molecules that carried VPg^mut^ at their 5’end. A pathway that could possibly lead to wild type infection can be seen in the way that a few of these VPg^mut^-linked PVA^WT^ RNA molecules fended the antiviral defense and became associated with polysomes and replication complexes to produce wild type VPg and wild type VPg-linked progeny RNAs, respectively. Since we could not verify the onset of wild type infection by particle formation in the local leaves, we assume that reversion occurred only in few scattered cells per infiltrated area. The spread of the reverted PVA in the infiltrated leaves may become limited by ongoing PVA^VPgmut^ expression in the neighboring cells. However, the emergence of particles and the onset of full scale PVA^WT^ infection in the systemic leaves proved that PVA^VPgmut^ RNA was capable of producing a low quantity of PVA^WT^ viruses in local leaves, although VPg lacked the YTDIRLI motif for eIF4E binding in the beginning. 

In spite of the fact that PVA RNA could replicate in the presence of the eIF4E-binding deficient VPg^mut^, the infection reached neither the wild type level of viral gene expression nor RNA accumulation. This may mean that the VPg^mut^-linked PVA RNA has difficulty engaging with the polysomes and new rounds of replication as PVA^WT^ RNA does. PVA VPg, when overexpressed with PVA RNA, enhances both the accumulation of viral RNA and the expression of 3’RLUC [35,54]. This phenomenon takes place both for replicating VPg-linked RNA and non-replicating capped PVA^ΔGDD^ RNAs. Therefore, it is a consequence of RNA stabilization rather than enhanced replication. Interestingly, VPg^mut^ overexpression was able to enhance the RNA and 3’RLUC accumulation of the capped PVA^ΔGDD^ RNA (see Figure 4) but not that of the VPg-linked PVA^WT^ RNA. This suggests that the stability of the PVA RNA appearing from the replication complex could not be supported by VPg^mut^, as with VPg, and the defect in the VPg-eIF(iso)4E interaction may have played a role here. In this assay, PVA^VPgmut^ RNA behaved like the non-replicating capped PVA^ΔGDD^ RNA. In fact, a substantial amount of the transcripts in these cells were capped ones that were exported from the nucleus, as the viral RNA amount in PVA^VPgmut^-expressing cells remained close to that of PVA^ΔGDD^ RNA (see Figure 2B).

We previously reported that PVA infection induces the formation of granules, PGs, which contain viral RNA and several host and viral RNA-binding proteins [37]. We demonstrated that the silencing suppressor protein HCPro is the sole PVA component that is responsible for the induction of PGs and proposed that PG formation is related to the protection of viral RNA from RNA silencing. Acidic ribosomal protein P0, which contributes to PVA gene expression [47], is one of the PG proteins. When this protein was used as the reporter of PG formation, we observed that in the presence of the VPg^mut^ protein, the number of PGs increased. The increase was observed regardless of whether VPg^mut^ was expressed from PVA RNA in cis or from an expression construct in trans (see Figure 5). In TuMV infection, TuMV HCPro that is associated with PGs is targeted for degradation by autophagy unless it is protected by TuMV VPg [38]. In [38], the authors proposed that autophagy of PGs is a second layer of a plant’s antiviral defense, RNA silencing being the first one. PG accumulation in the presence of VPg^mut^ may suggest that the mutations in the eIF(iso)4E-binding site do not affect VPg’s capacity to inhibit autophagy. 

The PGs observed in the presence of VPg^mut^ may not have been similar to those formed during wild type infection. When the localization of PVA RNA in PGs was tested, a reduction was observed in the presence of VPg^mut^ compared to that of the wild type VPg. In the test, we used PVA^ΔGDD^ RNA that was tagged with several copies of the B-box sequence, which binds the λ22 peptide [55]. The non-replicating RNA was used because the insertion of several copies of the B-box element to 3’UTR of PVA^WT^ may compromise infection. We demonstrated the presence of PVA^ΔGDD^ RNA in PGs with this methodology in [37]. The significantly reduced amount of co-localization between PVA^ΔGDD^ RNA and P0 in PGs of the cells that express VPg^mut^ indicated that VPg^mut^ negatively affected the transport of PVA^ΔGDD^ RNA in to the P0-containing PGs. The question of whether replicating PVA RNA would behave similarly to the capped PVA^ΔGDD^ RNA in this respect remains. Protection from RNA silencing is especially important for PVA^WT^ RNA, as the replicating viruses are strong inducers of RNA silencing in planta [58]. According to our hypothesis, the central function of PG formation in PVA^WT^ infection is the suppression of RNA silencing [37]. On these lines, the interrupted VPg-eIF(iso)4E interaction and the resulting reduced transfer of PVA RNA to PGs could expose PVA RNA to degradation by RNA silencing. This study provides three lines of evidence in support of this: 1) PVA RNA did not accumulate in PVA^VPgmut^ plants in spite of the capped PVA^VPgmut^ RNA being replicated; 2) there was a significant reduction of PVA^ΔGDD^ RNA in PGs in the presence of VPg^mut^; and 3) VPg^mut^ was not able to increase PVA^WT^ RNA accumulation similarly to the wild type VPg. In accordance with this, the interactions between VPg and HCPro with eIF4E/(iso)4E were both suggested in [44] to be of importance for the suppression of RNA silencing. 

Though PVA RNA is associated with PGs, it is not needed for their formation [37]. HCPro is the sole requirement, and, in the absence of PVA RNA, the PG-like structures likely still contain HCPro and its interactors. This study showed that the PG-like aggregates contained, in addition to the P0 protein, a substantial amount of eIF(iso)4E in VPg^mut^-expressing cells. An interesting network of binding sites was demonstrated in [45], in which PVA VPg binds to HCPro and to two binding sites in eIF4E/(iso)4E, one of which is shared with HCPro (the binding domain for proteins that carry the YXXXXLΦ motif). The abundant presence of eIF(iso)4E in PGs in spite of VPg^mut^ may arise from the interaction of eIF(iso)4E with HCPro [43,45]. VPg is responsible for channeling PVA RNA to translation [37], a function that requires eIF(iso)4E, VPg and PVA RNA [35]. In PVA^WT^ infection, enhanced channeling is seen as the dispersal of PGs [37]. The failure of VPg^mut^ to disperse PGs may be the reason for the accumulation of the PG-like aggregates seen in Figure 5. There are many open questions related to these complex processes. Among them is the question about the exact roles of genome-linked and free cytoplasmic VPg in organizing the eIF4E and other required interactions. Another question is whether PVA RNA goes to translation via PGs or it ends up with PGs when there is not enough VPgs to channel it to translation. Nevertheless, many of the same proteins that co-localize to PGs have a role in PVA translation along with VPg [37]. 

In [59], we detected HCPro-containing high molecular weight complexes in association with polysomes in PVA-infected plants and proposed that a complex consisting of a genome-linked VPg, a free cytoplasmic VPg, HCPro, the CI protein, eIF(iso)4E, eIF4A, eIF4G and AGO1 may form around the 5’UTR of PVA RNA to assist viral translation. The suggestion is based on the knowledge of the proteins participating in PVA translation [35,37,47], the proteomic data of HCPro interactors published in [59] and in the PRIDE repository [60], with the dataset identifier PXC016349, Western blot data of HCPro-associated polysomes [59], and potyviral literature on VPg, HCPro, CI, eIF4E and eIF4G interactions [32,45,51,61,62]. An interesting future line of investigation will be to understand if the viral high molecular weight complex ending up with polysomes in PVA-infected cells contributes to the successful transportation of PVA RNA the whole way from replication, via the silencing suppression pathway, to translation. 

As a testable future model, we propose that an essential function of eIF(iso)4E-VPg binding is to protect PVA RNA from being sent to the RNA silencing pathway. We suggest that the VPg-eIF(iso)4E interaction is required for the successful assembly of a protective protein complex around PVA RNA, thus enabling its transfer to the RNA silencing suppression pathway, which involves PGs as one component, and further to polysomes for viral protein synthesis. 

## References

[1] Scholthof K.B., Adkins S., Czosnek H., Palukaitis P., Jacquot E., Hohn T., Hohn B., Saunders K., Candresse T., Ahlquist P. (2011). Top 10 plant viruses in molecular plant pathology. Mol. Plant Pathol..

[2] Topisirovic I., Sonenberg N. (2011). Translational control by the eukaryotic ribosome. Cell.

[3] Browning K.S. (2004). Plant translation initiation factors: It is not easy to be green. Biochem. Soc. Trans..

[4] Gingras A.C., Raught B., Sonenberg N. (1999). eIF4 initiation factors: effectors of mRNA recruitment to ribosomes and regulators of translation. Annu. Rev. Biochem..

[5] Rajamäki M.L., Valkonen J.P. (1999). The 6K2 protein and the VPg of *potato virus A* are determinants of systemic infection in *Nicandra physaloides*. Mol. Plant Microbe Interact..

[6] Rajamäki M.L., Valkonen J.P. (2002). Viral genome-linked protein (VPg) controls accumulation and phloem-loading of a potyvirus in inoculated potato leaves. Mol. Plant Microbe Interact..

[7] Léonard S., Plante D., Wittmann S., Daigneault N., Fortin M.G., Laliberté J.F. (2000). Complex formation between potyvirus VPg and translation eukaryotic initiation factor 4E correlates with virus infectivity. J. Virol..

[8] Borgstrøm B., Johansen I.E. (2001). Mutations in pea seedborne mosaic virus genome-linked protein VPg after pathotype-specific virulence in *Pisum sativum*. Mol. Plant Microbe Interact..

[9] Moury B., Morel C., Johansen E., Guilbaud L., Souche S., Ayme V., Caranta C., Palloix A., Jacquemond M. (2004). Mutations in *potato virus Y* genome-linked protein determine virulence toward recessive resistances in *Capsicum annuum* and *Lycopersicon hirsutum*. Mol. Plant Microbe Interact..

[10] Gallois J.L., Charron C., Sánchez F., Pagny G., Houvenaghel M.C., Moretti A., Ponz F., Revers F., Caranta C., German-Retana S. (2010). Single amino acid changes in the *turnip mosaic virus* viral genome-linked protein (VPg) confer virulence towards *Arabidopsis thaliana* mutants knocked out for eukaryotic initiation factors eIF(iso)4E and eIF(iso)4G. J. Gen. Virol..

[11] Perez K., Yeam I., Kang B.C., Ripoll D.R., Kim J., Murphy J.F., Jahn M.M. (2012). *Tobacco etch virus* infectivity in Capsicum spp. is determined by a maximum of three amino acids in the viral virulence determinant VPg. Mol. Plant Microbe Interact..

[12] Wang A., Krishnaswamy S. (2012). Eukaryotic translation initiation factor 4E-mediated recessive resistance to plant viruses and its utility in crop improvement. Mol. Plant Pathol..

[13] Robaglia C., Caranta C. (2006). Translation initiation factors: A weak link in plant RNA virus infection. Trends Plant Sci..

[14] Truniger V., Aranda M.A. (2009). Recessive resistance to plant viruses. Adv. Virus Res..

[15] Okade H., Fujita Y., Miyamoto S., Tomoo K., Muto S., Miyoshi H., Natsuaki T., Rhoads R.E., Ishida T. (2009). *Turnip mosaic virus* genome-linked protein VPg binds C-terminal region of cap-bound initiation factor 4E orthologue without exhibiting host cellular specificity. J. Biochem..

[16] Sato M., Nakahara K., Yoshii M., Ishikawa M., Uyeda I. (2005). Selective involvement of members of the eukaryotic initiation factor 4E family in the infection of *Arabidopsis thaliana* by potyviruses. FEBS Lett..

[17] Nicaise V., Gallois J.L., Chafiai F., Allen L.M., Schurdi-Levraud V., Browning K.S., Candresse T., Caranta C., Le Gall O., German-Retana S. (2007). Coordinated and selective recruitment of eIF4E and eIF4G factors for potyvirus infection in *Arabidopsis thaliana*. FEBS Lett..

[18] Duprat A., Caranta C., Revers F., Menand B., Browning K.S., Robaglia C. (2002). The *Arabidopsis* eukaryotic initiation factor (iso)4E is dispensable for plant growth but required for susceptibility to potyviruses. Plant J..

[19] Nicaise V., German-Retana S., Sanjuán R., Dubrana M.P., Mazier M., Maisonneuve B., Candresse T., Caranta C., Le Gall O. (2003). The eukaryotic translation initiation factor 4E controls lettuce susceptibility to the Potyvirus *Lettuce mosaic virus*. Plant Physiol..

[20] Jenner C.E., Nellist C.F., Barker G.C., Walsh J.A. (2010). *Turnip mosaic virus* (TuMV) is able to use alleles of both eIF4E and eIF(iso)4E from multiple loci of the diploid *Brassica rapa*. Mol. Plant Microbe Interact..

[21] Sanfaçon H. (2015). Plant Translation Factors and Virus Resistance. Viruses.

[22] Hashimoto M., Neriya Y., Yamaji Y., Namba S. (2016). Recessive Resistance to Plant Viruses: Potential Resistance Genes Beyond Translation Initiation Factors. Front. Microbiol..

[23] Shopan J., Mou H., Zhang L., Zhang C., Ma W., Walsh J.A., Hu Z., Yang. J., Zhang M. (2017). Eukaryotic translation initiation factor 2B-beta (eIF2Bβ), a new class of plant virus resistance gene. Plant J..

[24] Charron C., Nicolaï M., Gallois J.L., Robaglia C., Moury B., Palloix A., Caranta C. (2008). Natural variation and functional analyses provide evidence for co-evolution between plant eIF4E and potyviral VPg. Plant J..

[25] Nicaise V. (2014). Crop immunity against viruses: outcomes and future challenges. Front. Plant Sci..

[26] Moury B., Charron C., Janzac B., Simon V., Gallois J.L., Palloix A., Caranta C. (2014). Evolution of plant eukaryotic initiation factor 4E (eIF4E) and potyvirus genome-linked protein (VPg): a game of mirrors impacting resistance spectrum and durability. Infect. Genet. Evol..

[27] Monzingo A.F., Dhaliwal S., Dutt-Chaudhuri A., Lyon A., Sadow J.H., Hoffman D.W., Robertus J.D., Browning K.S. (2007). The structure of eukaryotic translation initiation factor-4E from wheat reveals a novel disulfide bond. Plant. Physiol..

[28] Walter J., Charon J., Hu Y., Lachat J., Leger T., Lafforgue G., Barra A., Michon T. (2019). Comparative analysis of mutational robustness of the intrinsically disordered viral protein VPg and of its interactor eIF4E. PLoS ONE.

[29] Bruun-Rasmussen M., Møller I.S., Tulinius G., Hansen J.K., Lund O.S., Johansen I.E. (2007). The same allele of translation initiation factor 4E mediates resistance against two Potyvirus spp. in *Pisum sativum*. Mol. Plant Microbe Interact..

[30] Coutinho de Oliveira L., Volpon L., Rahardjo A.K., Osborne M.J., Culjkovic-Kraljacic B., Trahan C., Oeffinger M., Kwok B.H., Borden K.L.B. (2019). Structural studies of the eIF4E-VPg complex reveal a direct competition for capped RNA: Implications for translation. Proc. Natl. Acad. Sci. USA.

[31] Puurand U., Valkonen J.P., Mäkinen K., Rabenstein F., Saarma M. (1996). Infectious in vitro transcripts from cloned cDNA of the potato A potyvirus. Virus Res..

[32] Michon T., Estevez Y., Walter J., German-Retana S., Le Gall O. (2006). The potyviral virus genome-linked protein VPg forms a ternary complex with the eukaryotic initiation factors eIF4E and eIF4G and reduces eIF4E affinity for a mRNA cap analogue. FEBS J..

[33] Khan M.A., Miyoshi H., Gallie D.R., Goss D.J. (2008). Potyvirus genome-linked protein, VPg, directly affects wheat germ in vitro translation: interactions with translation initiation factors eIF4F and eIFiso4F. J. Biol. Chem..

[34] Miyoshi H., Okade H., Muto S., Suehiro N., Nakashima H., Tomoo K., Natsuaki T. (2008). *Turnip mosaic virus* VPg interacts with *Arabidopsis thaliana* eIF(iso)4E and inhibits in vitro translation. Biochimie.

[35] Eskelin K., Hafrén A., Rantalainen K.I., Mäkinen K. (2011). Potyviral VPg enhances viral RNA Translation and inhibits reporter mRNA translation in planta. J. Virol..

[36] Eskelin K., Suntio T., Hyvärinen S., Hafren A., Mäkinen K. (2010). Renilla luciferase-based quantitation of Potato virus A infection initiated with *Agrobacterium* infiltration of *N. benthamiana* leaves. J. Virol. Methods.

[37] Hafrén A., Lõhmus A., Mäkinen K. (2015). Formation of Potato Virus A-Induced RNA Granules and Viral Translation Are Interrelated Processes Required for Optimal Virus Accumulation. PLoS Pathog..

[38] Hafrén A., Hofius D. (2017). NBR1-mediated antiviral xenophagy in plant immunity. Autophagy.

[39] Tavert-Roudet G., Anne A., Barra A., Chovin A., Demaille C., Michon T. (2017). The Potyvirus Particle Recruits the Plant Translation Initiation Factor eIF4E by Means of the VPg covalently Linked to the Viral RNA. Mol. Plant Microbe Interact..

[40] Lellis A.D., Kasschau K.D., Whitham S.A., Carrington J.C. (2002). Loss-of-susceptibility mutants of *Arabidopsis thaliana* reveal an essential role for eIF(iso)4E during potyvirus infection. Curr. Biol..

[41] Contreras-Paredes C.A., Silva-Rosales L., Daròs J.A., Alejandri-Ramírez N.D., Dinkova T.D. (2013). The absence of eukaryotic initiation factor eIF(iso)4E affects the systemic spread of a *Tobacco etch virus* isolate in *Arabidopsis thaliana*. Mol. Plant Microbe Interact..

[42] Marcotrigiano J., Gingras A.C., Sonenberg N., Burley S.K. (1999). Cap-dependent translation initiation in eukaryotes is regulated by a molecular mimic of eIF4G. Mol. Cell.

[43] Ala-Poikela M. (2014). Coordinated functions of the HCpro and VPg proteins of Potato virus A (PVA) with the translation initiation factors eIF4E and eIF(iso)4E. PhD Thesis.

[44] Ala-Poikela M., Goytia E., Haikonen T., Rajamäki M.L., Valkonen J.P.T. (2011). Helper component proteinase of the genus Potyvirus is an interaction partner of translation initiation factors eIF(iso)4E and eIF4E and contains a 4E binding motif. J. Virol..

[45] Ala-Poikela M., Rajamäki M.L., Valkonen J.P.T. (2019). A Novel Interaction Network Used by Potyviruses in Virus-Host Interactions at the Protein Level. Viruses.

[46] Puurand U., Mäkinen K., Paulin L., Saarma M. (1994). The nucleotide sequence of potato virus A genomic RNA and its sequence similarities with other potyviruses. J. Gen. Virol..

[47] Hafrén A., Eskelin K., Mäkinen K. (2013). Ribosomal protein P0 promotes Potato virus A infection and functions in viral translation together with VPg and eIF(iso)4E. J. Virol..

[48] Liu D., Shi L., Han C., Yu J., Li D., Zhang Y. (2012). Validation of reference genes for gene expression studies in virus infected *Nicotiana benthamiana* using quantitative real-time PCR. PLoS ONE.

[49] Rhoads R.E. (2009). eIF4E: New family members, new binding partners, new roles. J. Biol. Chem..

[50] Puustinen P., Mäkinen K. (2004). Uridylylation of the potyvirus VPg by viral replicase NIb correlates with the nucleotide binding capacity of VPg. J. Biol. Chem..

[51] Oruetxebarria I., Guo D., Merits A., Mäkinen K., Saarma M., Valkonen J.P.T. (2001). Identification of the genome-linked protein in virions of *Potato virus A*, with comparison to other members in genus *Potyvirus*. Virus Res..

[52] Valli A., Gallo A., Calvo M., de Jesús Pérez J., García J.A. (2014). A novel role of the potyviral helper component proteinase contributes to enhance the yield of viral particles. J. Virol..

[53] Gallo A., Valli A., Calvo M., García J.A. (2018). A functional link between RNA replication and virion assembly in the Potyvirus. J. Virol..

[54] Saha S., Hafren A., Mäkinen K. (2019). Dynamics of Protein Accumulation from the 3’ End of Viral RNA are different from those in the rest of the genome in Potato Virus A infection. J. Virol..

[55] Schönberger J., Hammes U.Z., Dresselhaus T. (2012). In vivo visualization of RNA in plants cells using the λN₂₂ system and a GATEWAY-compatible vector series for candidate RNAs. Plant J..

[56] Anindya R., Chittori S., Savithri H.S. (2005). Tyrosine 66 of Pepper vein banding virus genome-linked protein is uridylylated by RNA-dependent RNA polymerase. Virology.

[57] Paul A.V., Wimmer E. (2015). Initiation of protein-primed picornavirus RNA synthesis. Virus Res..

[58] Pumplin N., Voinnet O. (2013). RNA silencing suppression by plant pathogens: defense, counter-defense and counter-counter-defense. Nat. Rev. Microbiol..

[59] Ivanov K.I., Eskelin K., Bašić M., De S., Lõhmus A., Varjosalo M., Mäkinen K. (2016). Molecular insights into the function of the viral RNA silencing suppressor HC-pro. Plant J..

[60] Perez-Riverol Y., Csordas A., Bai J., Bernal-Llinares M., Hewapathirana S., Kundu D.J., Inuganti A., Griss J., Mayer G., Eisenacher M. (2019). The PRIDE database and related tools and resources in 2019: improving support for quantification data. Nucleic Acids Res..

[61] Roudet-Tavert G., Michon T., Walter J., Delaunay T., Redondo E., Le Gall O. (2007). Central domain of a potyvirus VPg is involved in the interaction with the host translation initiation factor eIF4E and the viral protein HcPro. J. Gen. Virol..

[62] Tavert-Roudet G., Abdul-Razzak A., Doublet B., Walter J., Delaunay T., German-Retana S., Michon T., Le Gall O., Candresse T. (2012). The C terminus of lettuce mosaic potyvirus cylindrical inclusion helicase interacts with the viral VPg and with lettuce translation eukaryotic initiation factor 4E. J. Gen. Virol..

